# Elucidating molecular pathogenesis and developing targeted therapeutic interventions for cerebrovascular endothelial cell-mediated vascular dementia

**DOI:** 10.3389/fnagi.2025.1623050

**Published:** 2025-07-16

**Authors:** Yanrong Yang, Hua Chen, Qibing Liu, Yang Niu, Chunyang Mao, Rui Wang

**Affiliations:** ^1^College of Pharmaceutical Science and Key Laboratory of Ministry of Education in Protection and Utilization of Medicinal Resources of Liupanshan Area, Ningxia Medical University, Yinchuan, Ningxia, China; ^2^Engineering Research Center of Tropical Medicine Innovation and Translational of Hainan and Department of Pharmacy, The First Affiliated Hospital of Hainan Medical University, Haikou, Hainan, China; ^3^Key Laboratory of Ministry of Education in Modernization of Minority Medicine, Ningxia Medical University, Yinchuan, Ningxia, China

**Keywords:** vascular dementia, neurovascular coupling, cerebrovascular endothelial cells, molecular mechanisms, treatment and strategy

## Abstract

Vascular dementia (VaD) ranks as the second most prevalent subtype of dementia, surpassed only by Alzheimer’s disease (AD). The maintenance of neurological function and cerebral homeostasis critically depends on precisely regulated blood flow within the intricately organized cerebrovascular network. Disruptions in cerebral hemodynamics may impair neurovascular homeostasis, thereby inducing pathophysiological cascades characterized by oxidative stress, neuroinflammation, and neuronal degeneration. Emerging evidence identifies cerebrovascular dysregulation and impaired neurovascular coupling (NVC) as primary pathogenic mechanisms underlying VaD, emphasizing the necessity to elucidate their complex interplay. Cerebrovascular endothelial cells exhibit remarkable heterogeneity, serving dual roles as both architectural components of the blood–brain barrier (BBB) and functional regulators of NVC. Furthermore, pericytes residing abluminal on capillary endothelia demonstrate critical involvement in hemodynamic modulation through contractile regulation of microvascular tone, while concurrently maintaining BBB integrity through dynamic paracrine signaling. This study examines cerebrovascular endothelial-neuronal interactions within the neurovascular unit (NVU) framework, analyzing their bidirectional regulatory mechanisms and therapeutic potential in cognitive dysfunction remediation. The pathophysiological progression of VaD manifests through multiple interdependent pathways, including cerebral hypoperfusion, oxidative stress cascades, neuroinflammatory responses, mitochondrial dysregulation, and electrolyte homeostasis perturbations. Through three interventional axes: (1) BBB fortification strategies; (2) cerebral hemodynamic optimization and NVC enhancement; (3) nanotherapeutic platforms integrating endothelial-specific molecular targets we systematically evaluate endothelial-centric therapeutic paradigms. This multi-modal approach proposes novel mechanistic insights and clinical translation frameworks for VaD management.

## 1 Introduction

Vascular dementia (VaD) is a form of dementia characterized by cognitive impairment resulting from disrupted cerebral blood flow (CBF) and global or localized vascular lesions in the brain (Morgan and Mc Auley, 2024; Tiwari et al., 2021). As a subtype of dementia, it is regarded as one of the most severe forms of vascular cognitive impairment (VCI) (Custodero et al., 2022). Among dementia subtypes, VaD ranks second in prevalence after Alzheimer’s disease (AD), accounting for approximately 25% of all dementia cases (Chan et al., 2013; Jia et al., 2020). Globally, the overall prevalence of dementia in adults aged 65 years and older is higher in women than in men (Huque et al., 2023). In Finland, the annual incidence of dementia with a vascular component was reported to be 81.4 per 100,000 individuals, with 23.8% of dementia cases being associated with vascular disease (Tuominen et al., 2025). The global prevalence of dementia is projected to triple by 2050 (Zhu et al., 2022). Aging populations and increased life expectancy are contributing factors to the rising burden of disease, as well as the complexities and uncertainties involved in dementia management. From the perspective of cerebrovascular endothelial cells, this review provides an in-depth analysis of the pathogenesis of VaD and evaluates current potentially valuable treatment approaches and strategies.

## 2 The mechanisms underlying cerebrovascular endothelia in the regulation of NVC: a comprehensive review

### 2.1 Cerebrovascular endothelial cells constitute part of the NVU and participate in the BBB

**Figure 1:**
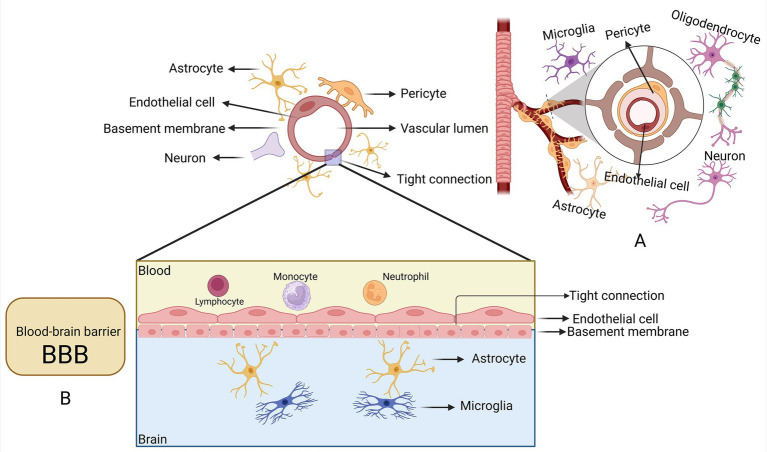
Schematic representation of the BBB and NVU. **(A)** Within the NVU, vascular endothelial cells are enveloped by the terminal endfeet of astrocytes. Both pericytes and astrocytes interact with and connect various cell types in this complex structure. **(B)** The BBB, positioned at the core of the NVU, is constituted by a continuous layer of endothelial cells lining the blood vessels. In the BBB, tight junctions formed by endothelial cells strictly regulate the transport of ions, molecules, and cells between the blood and the brain. The function of these tight junctions is modulated by surrounding cells, including pericytes, astrocytes, perivascular oligodendrocyte precursor cells (OPCs), interneurons, perivascular macrophages, and microglia. Pericytes reside on the luminal surface of the endothelium and are embedded within the basement membrane. Astrocytes undergo a process of spreading and polarization, nearly fully encapsulating the blood vessels. Created in https://BioRender.com.

VaD is frequently associated with damage to cerebrovascular endothelial cells (Goncharov et al., 2024). The concept of the neurovascular unit (NVU) is introduced to highlight the intricate relationship between brain cells and the cerebrovascular system (Iadecola, 2017). The NVU comprises neurons, glial cells, pericytes, endothelial cells, and vascular smooth muscle cells. As shown in Figure 1. From a functional perspective, the dynamic interactions among these cellular components contribute to the precise regulation of CBF, the maintenance of blood–brain barrier (BBB) integrity, the fulfillment of metabolic demands, and the facilitation of brain development, nutrition, and repair. Cerebrovascular endothelial cells enable the selective passage of nutrients and molecules essential for brain function while preventing the ingress of neurotoxic substances (Alkhalifa et al., 2023; Montagne et al., 2017). The NVU ensures the proper functioning of neuronal circuits by regulating BBB permeability, modulating CBF, and preserving the stability of the neuronal microenvironment (Smith et al., 2022). Consequently, the heterogeneity of cerebrovascular endothelial cells not only constitutes a major structural component of the BBB but also serves as a critical functional link in neural coupling activity.

### 2.2 Cerebrovascular endothelial cells play a critical role in the regulation of NVC

The cerebrovascular system supplies energy for cerebral neural activity, and fluctuations in neural activity elicit corresponding alterations in regional CBF within seconds, a mechanism formally termed neurovascular coupling (NVC). The precise interdependency between neuronal activity and CBF substantiates the brain’s minimal metabolic reserves and the imperative for rapid oxygen and glucose mobilization to activated cerebral regions CBF modulation is mediated through NVC mechanisms orchestrated by specialized NVU (Banerjee and Banerjee, 2024). Compromised NVC functionality may precipitate cerebral microcirculatory ischemia and hypoxia, impair local neuronal signal propagation, exacerbate cerebral small vessel pathology, and ultimately manifest as cognitive impairment or dementia (Chow et al., 2020). NVC constitutes a fundamental regulatory framework for cerebral hemodynamics. Age-related deterioration of this mechanism may constitute a pathogenic pathway toward cognitive decline (Tarantini et al., 2019). Vascular endothelial cells, recognized as key effectors in hemodynamic regulation through chemical and mechanical transduction, have emerged as critical components in NVC research (Harraz et al., 2022).

#### 2.2.1 Regulation of neuronal activity on cerebrovascular endothelial cells in NVC

**Figure 2:**
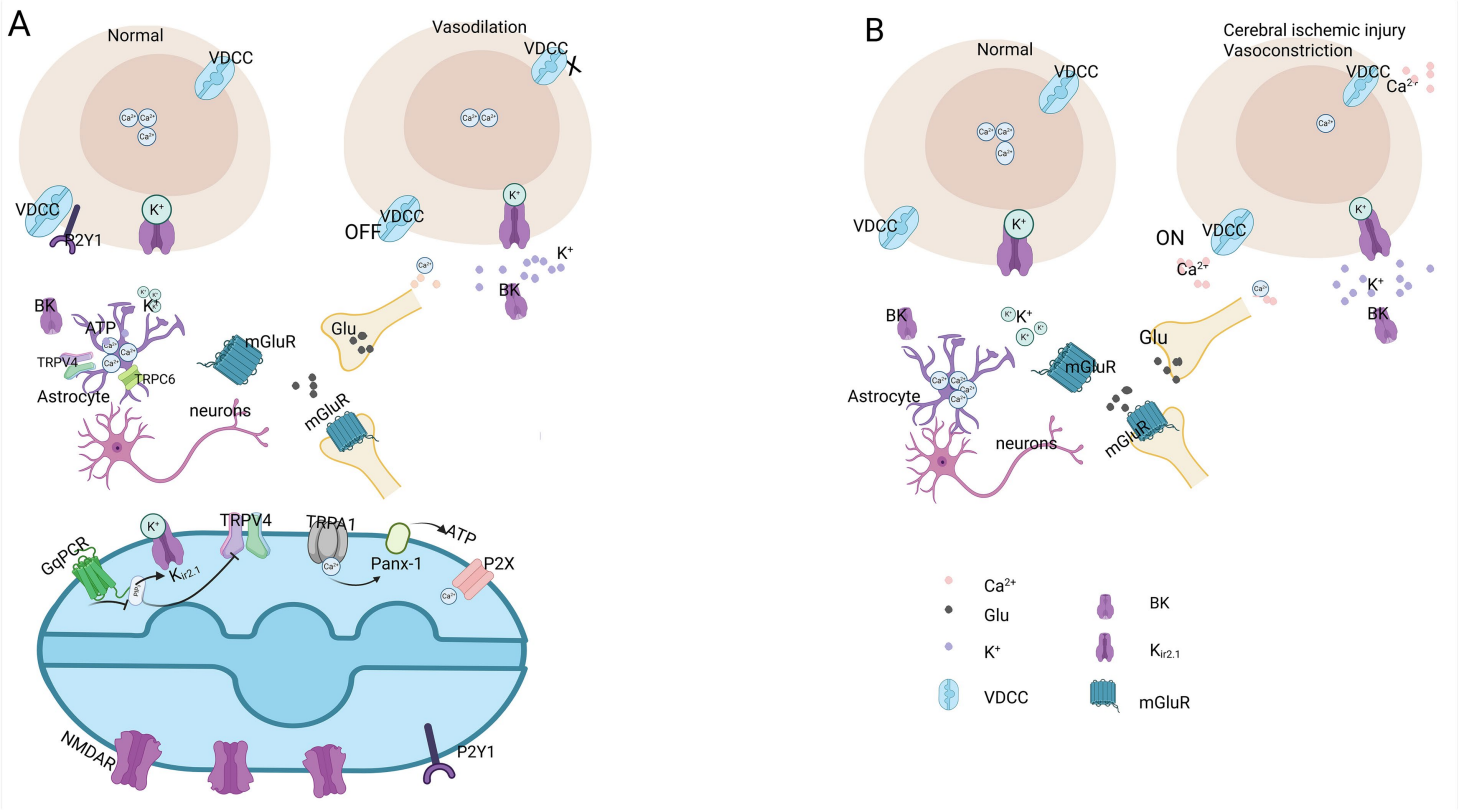
Schematic illustration of ion channel alterations in endothelial cells during NVC under normal brain function compared to the ischemic injury state. **(A)** In normal brain function, vasodilation occurs during neuronal activity. Voltage-dependent calcium channels (VDCCs) on the cell membrane remain closed, leading to a reduction in intracellular calcium ions, an increase in extracellular potassium ions, and a decrease in interneuronal glutamate (Glu). Ca^2+^ influx through TRPV4 channels is promoted by Gq protein-coupled receptor (GqPCR) signaling in the endothelium. GqPCR signaling disinhibits TRPV4 channels and lowers the activity of Kir channels. Ca^2+^ influx signals through TRPA1 channels stimulate nearby Pannexin 1 channels, promoting ATP release ATP activates Ca^2+^-permeable purinergic P2X receptor ion channels. **(B)** In ischemic brain injury, vasoconstriction occurs during neuronal activity. The entire cell undergoes shrinkage, voltage-dependent calcium channels on the cell membrane open, causing an increase in intracellular calcium ions and extracellular potassium ions, as well as an elevation in interneuronal glutamate levels. Via the up-regulation of functional Kir2.1 expression and presumably enhances Ca^2+^ entry. Glu: glutamate; VDCC: Voltage-dependent calcium channel; BK: large-conductance calcium-activated potassium channel; K_ir_: inward rectifying potassium channel; mGluR: metabotropic glutamate receptor. Created in https://BioRender.com.

Normal brain function relies on the precise coupling between the NVU, neuronal activity, and vascular function. NVC involves the localized activation of neuronal activity, which subsequently increases regional CBF to efficiently deliver nutrients and clear metabolic waste (Kaplan et al., 2020). In neurons, the arrival of the excitatory neurotransmitter glutamate at the presynaptic membrane triggers the activation of postsynaptic NMDA receptors and AMPA receptors. This cascade leads to increased calcium influx, the subsequent activation of calcium-dependent enzymes such as nitric oxide (NO) synthase and cyclooxygenase-2 (COX-2), the release of NO and prostaglandins, and ultimately the dilation of small blood vessels, thereby enhancing blood flow (Schaeffer and Iadecola, 2021). As depicted in Figure 2, neurons modulate CBF by releasing signaling molecules such as glutamate and Ca^2+^. This signaling elicits vascular responses either directly or indirectly via associated cellular mechanisms. Astrocytes play a pivotal role in transmitting neuronal activity signals to the local vasculature. Positioned closely to synapses and local microvessels, astrocytes bridge neural activity with microvascular function. Glutamate acts on astrocytic receptors, leading to an elevation in intracellular calcium concentration, which subsequently activates phospholipase A2 (PA2) and triggers the release of arachidonic acid. Prostaglandin E2 (PE2) and Epoxyeicosatrienoic acids (EETs) are generated through the cyclooxygenase and cytochrome P450 enzymatic pathways, respectively, inducing arteriolar dilation (McConnell et al., 2019). Another plausible mechanism involves a rapid increase in intracellular Ca^2+^ concentration within astrocytes, which activates large-conductance Ca^2+^-activated K^+^ channels, resulting in K^+^ efflux and subsequent vascular smooth muscle relaxation (Iadecola, 2017). Additionally, ATP secreted by astrocytes binds to P2Y1 receptors on endothelial cells, activating endothelial nitric oxide synthase (eNOS) and prompting endothelial cells to produce NO, thereby causing vasodilation (da Silva et al., 2009).

#### 2.2.2 Cerebral vascular endothelial cell receptors, their secretory functions, and the regulation of neurons in NVC mechanisms

Endothelial cells play a critical role in the regulation of cerebral vascular tone. In addition to modulating NVC responses through the expression of eNOS and the production of NO, endothelial cells also possess a variety of ion channels, such as the inwardly rectifying potassium channel 2.1 (K_ir2.1_), N-methyl-D-aspartate receptors (NMDARs), and Transient receptor potential ankyrin 1 (TRPA1) channel. These channels enable endothelial cells to detect neuron-derived signals and initiate vasodilation. Neuronal activity elevates the extracellular potassium (K^+^) concentration, activating the K_ir2.1_ channel on endothelial cells. This activation promotes intracellular K^+^ efflux, leading to hyperpolarization of capillary endothelial cells and adjacent pericytes via myo-endothelial gap junctions. Endothelium-dependent hyperpolarization preferentially propagates upstream through endothelial gap junctions, inducing smooth muscle cell hyperpolarization and subsequent arteriolar relaxation, thereby enhancing regional CBF (Moccia et al., 2022). Loss of endothelial NMDAR function resulted in a 50% reduction in CBF increase during whisker stimulation, indicating that the absence of NMDAR substantially impairs NVC responses (Hogan-Cann et al., 2019). Activation of TRPA1 channels in endothelial cells triggers the release of ATP via Pannexin-1 (Panx-1) channels, which subsequently acts on P2X receptors in neighboring cells to generate a slowly propagating intercellular Ca^2+^ signal within the brain capillary endothelium. Upon reaching the post arteriolar transition zone, this Ca^2+^ signal is converted into rapidly propagating electrical signals by small and intermediate conductance potassium (K^+^) channels (SK and IK). Activation of these channels leads to hyperpolarization of the endothelial membrane, which is then transmitted through gap junctions to arteriolar smooth muscle cells, thereby inducing vasodilation (Thakore et al., 2021).

The secretory functions of cerebrovascular endothelial cells and their regulatory roles in neuronal activity. Within the central nervous system, semaphoring 3G (Sema3G), synthesized by endothelial cells, enhances excitatory synaptic density by activating neural signaling pathways. Functioning as a vasculature-derived synaptic organizer, Sema3G modulates synaptic plasticity and facilitates hippocampus-dependent memory consolidation (Tan et al., 2019). Additionally, endothelial-derived Sema3G stimulates reparative angiogenesis through coordinated regulation of physiological vascular remodeling and suppression of pathological vascular degeneration (Chen et al., 2021). Metabolic byproducts of brain activity, such as adenosine, hydrogen ions, and lactate, may reverse the flow response (Freeman and Li, 2016). This may be the interaction mechanism of a feedback model.

#### 2.2.3 Pericyte-mediated regulation of NVC

Pericytes are integral cellular constituents of the NVU that participate in the precise regulation of CBF and vascular tone through capillary diameter modulation (Uemura et al., 2020). Experimental evidence demonstrates that pericytes depletion induces a pathological increase in non-perfused cerebral capillaries, a phenomenon driven by amplified leukocyte-endothelial cell interactions, degradation of the endothelial glycocalyx, and transcriptional upregulation of leukocyte adhesion molecules (Choe et al., 2022). Furthermore, astrocytes have been identified as essential contributors to NVC dynamics through their functional crosstalk with pericytes. 20-Hydroxyeicosatetraenoic acid (20-HETE), a key bioactive metabolite synthesized via the metabolism of arachidonic acid in astrocytes, facilitates CBF autoregulation through augmented pericytes contractility. Within the central nervous system (CNS), pericytes densely enwrap microvascular endothelial cells, thereby maintaining cerebrovascular barrier integrity. These mural cells exert regulatory control over capillary tone through coordinated microvascular constriction and relaxation, preserve BBB homeostasis, and contribute to neuroimmune surveillance functions. The pericyte-deficient murine model provides critical mechanistic insights into BBB compromise observed in dementia-related neurovascular pathology, demonstrating pericytes capacity to modulate endothelial transcriptional programming. While pericytes exhibit limited direct involvement in BBB-specific gene transcription, their synergistic interaction with endothelial cells in co-culture models markedly enhances the functional potency of the BBB (Procter et al., 2021). In the NVU, pericytes execute essential functions via orchestrated intercellular communication with adjacent cell populations, specifically endothelial cells, glial cells, and neurons. Pericytes dysfunction constitutes a critical etiological factor in multiple neuropathological conditions, encompassing cerebrovascular pathologies, acute ischemic stroke, AD spectrum disorders, and related neurological morbidities (Uemura et al., 2020). Within cerebrovascular disease paradigms, pericytes degeneration precipitates microvascular destabilization and myelin sheath degradation, whereas in the post-ischemic stroke milieu, pericyte-mediated vasoconstriction induces capillary-level perfusion deficits through the no-reflow phenomenon (Huang et al., 2020). Diminished pericytes density correlating with resultant microvascular compromise demonstrates a significant association with white matter volumetric loss and progressive cognitive dysfunction. Furthermore, pericyte depletion precipitates BBB integrity compromise, effectively impeding effective *β*-amyloid clearance and permitting neurotoxic substance extravasation into cerebral parenchymal tissues (Uemura et al., 2020).

#### 2.2.4 The role of neurovascular dysregulation in the pathogenesis of VaD

Neurovascular unit has emerged as a critical factor contributing to neuronal damage in ischemic brain diseases and neurodegenerative disorders. Elucidating the pathological mechanisms underlying abnormal NVC and their roles in the onset and progression of central nervous system diseases will offer novel strategies for clinical treatment and drug target identification. Dysfunction of the K_ir2.1_ ion channel in cerebrovascular endothelial cells is regarded as a potential contributor to NVU in patients. Reduced expression of K_ir2.1_ channel proteins in the brain and capillaries has been linked to NVU in a transgenic rodent model of AD (Lacalle-Aurioles et al., 2022; Taylor et al., 2022). Interventions that enhance mitochondrial antioxidant defenses exhibit significant protective effects on brain microvessels, thereby preserving NVC responses in aged mice (Csiszar et al., 2019). NO prevents endothelial cell senescence by maintaining the coupling of eNOS. The oxidation of tetrahydrobiopterin (BH4) plays a pivotal role in NO production mediated by eNOS (Hernandez-Navarro et al., 2024).

Diminished NO production is associated with brain microvascular endothelial dysfunction and NVU, potentially contributing to cognitive impairment (Tarantini et al., 2021b; Toth et al., 2015). Vascular-neuronal interactions are crucial for maintaining neural circuit robustness and sustaining cognitive performance. Within endothelial cells of the CNS, pericyte-derived vitronectin maintains blood-CNS barrier integrity through synergistic engagement with the integrin α5 receptor and ligand-receptor cross-talk among endothelial integrin receptors (Ayloo et al., 2022). Exosomes mediate critical regulatory functions in CNS homeostasis and pathology by facilitating intercellular transfer of bioactive cargos, encompassing lipids, structural proteins, messenger RNAs, and microRNAs. This mechanistic understanding has thus motivated emerging research exploring exosomal therapeutics as a viable neuroprotective strategy, which enhances endogenous neurogenesis to counteract CNS neuronal degeneration, thereby offering potential clinical applications for diverse neurodegenerative conditions (Yu et al., 2024).

## 3 The role of cerebrovascular endothelial dysfunction in the pathophysiological mechanisms of VaD

### 3.1 Cerebral vascular endothelial cells and ischemia and hypoxia

Cerebral hypoxia, either as global hypoxia or as focal cerebrovascular accident or stroke, is a severe neurological disorder with heterogeneous characteristics and multiple etiologies, which represents the second cause of death and the disease with the greatest sequelae of disability in the world. Although the hippocampus demonstrates heightened susceptibility to ischemic–hypoxic conditions, the vascular endothelial growth factor (VEGF) manifests enhanced resistance to hypoxic–ischemic neuronal injury. Quantification revealed significantly elevated densities of VEGF-immunopositivity cells in the hypoxic cerebral cortex relative to hippocampal regions (Jun et al., 2020). Compelling evidence indicates that chronic cerebral hypoperfusion (CCH) serves as a pivotal pathophysiological mechanism underlying the initiation and evolution of VaD (Chen et al., 2024). Prolonged ischemic–hypoxic stress induces degradation of BBB tight junction proteins, elevates microvascular permeability, and stimulates concomitant upregulation of pro-angiogenic mediators including VEGF-A within cerebral endothelial cells and pericytes. Experimental administration of minocycline in rodent models has been demonstrated to attenuate BBB compromise, ameliorate white matter pathology, and potentiate neovascularization processes (Yang et al., 2018). Hypoxia-inducible factor (HIF)-mediated signaling operates as an adaptive cytoprotective mechanism activated by hypoxia, ischemia, and related pathological states (Yang et al., 2013). Under hypoxic conditions, HIF-1*α* coordinates osteogenic and angiogenic processes through modulation of the VEGF/AKT/mTOR signaling axis, while simultaneously facilitating the recruitment of bone marrow-derived angiogenic progenitor cells to ischemic microenvironments (Song et al., 2023). Notably, HIF-1*α* demonstrates neuroprotective efficacy against acute ischemic and hypoxia-associated vascular dementia via coordinated activation of PI3K/AKT/mTOR and MAPK cascades (Abdel-Latif et al., 2018). Pharmacological interventions employing berberine have been shown to suppress pathological angiogenesis and cellular apoptosis through selective inhibition of the HIF-1*α*/VEGF/DLL-4/Notch-1 signaling nexus (Ai et al., 2022). Emerging evidence suggests that vascular endothelial cells exert neuroprotective influences during post-hypoxic neuronal pathophysiology, thereby positioning enhanced NVC dynamics and targeted vascular remodeling as promising therapeutic modalities for cerebral injury mitigation (Wu et al., 2016). Ischemia-induced degradation of the microvascular matrix architecture correlates temporally with microglial cathepsin L secretion during lesion progression. Strategic targeting of cathepsin L-mediated proteolytic activity and its synergistic interplay with matrix metalloproteinase (MMP) systems may offer novel approaches for attenuating microvascular compromise and hemorrhagic complications (Gu et al., 2015).

### 3.2 Cerebrovascular endothelial cells and their response to oxidative stress

Oxidative stress represents a pathological process characterized by the excessive accumulation of reactive oxygen species (ROS) and free radicals within the body, which can induce structural damage to cellular components. Naringenin mitigates oxidative stress in the hippocampus of vascular dementia rats by suppressing ROS production, reducing malondialdehyde (MDA) levels, and enhancing the enzymatic activities of superoxide dismutase (SOD) and glutathione peroxidase (GSH-Px) in the hippocampus. Furthermore, naringenin decreases the levels of pro-inflammatory cytokines while elevating those of anti-inflammatory cytokines in the hippocampus of 2-VO model rats, thereby alleviating hippocampal inflammatory responses in VaD model rats (Zhang et al., 2022). Oxidative stress may serve as a critical risk factor for Amyloid-beta (A*β*) deposition in the cerebrovascular system by initiating the amyloidogenic pathway of endothelial amyloid precursor protein (APP) processing. The increased *β*-secretase activity following oxidative stress exposure could potentially be mediated through the phosphorylation of p42/44 MAPK (Muche et al., 2017). Long-term administration of CU06-1004, a therapeutic agent targeting endothelial dysfunction through suppression of vascular leakage and enhancement of vascular integrity under ischemia–reperfusion conditions, markedly alleviated age-associated cerebral microvascular rarefaction and cerebrovascular senescence in aged murine models. Concurrent behavioral assessments revealed significant amelioration of motor coordination and cognitive performance in senescence-accelerated mice subjected to sustained CU06-1004 intervention (Kim et al., 2023). The neuroprotective efficacy of glycyrrhizin (GA) in rodent models of VaD was mechanistically linked to its dual capacity for attenuating oxidative stress and modulating voltage-gated Na^+^ channel activity within hippocampal CA1 pyramidal neurons. Collectively, these investigations furnish compelling preclinical evidence endorsing the therapeutic potential of glycyrrhizin in addressing cognitive impairments secondary to neurodegenerative disorders, cerebrovascular accidents, or neurotrauma (Guo et al., 2016). Beyond its chrono biotic functions, melatonin demonstrates substantial free radical-scavenging capabilities and exerts neuroprotection effects via mitigation of cerebral oxidative stress and suppression of neuroinflammatory cascades (Delgado-Lara et al., 2020). Notably, nicotinamide mononucleotide administration effectively enhances microvascular endothelial homeostasis and reinstates nitric oxide-dependent NVC mechanisms in aged murine subjects (Tarantini et al., 2019).

### 3.3 Cerebrovascular endothelial cells and their role in neuroinflammation

Neuroinflammation represents an immune cascade orchestrated by glial cells within the central nervous system, the principal site of innate immunity. CCH-induced persistent ischemia and hypoxia trigger excessive neuroinflammatory activation, culminating in cellular apoptosis, BBB compromise, and associated pathological manifestations that accelerate the pathogenesis of VaD (Tian et al., 2022). Notably, miR-193b-3p and miR-152-3p inhibition nullified tilianin-mediated cognitive enhancement achieved through p38-MAPK/NF-κB-p65 and Bcl2/Bax/Caspase-3/PARP pathway activation in 2-VO rat models. Crucially, calmodulin and Calmodulin-Stimulated Protein Kinase II (CaMKII) overexpression counteracted the synergistic therapeutic effects of tilianin and miR-193b-3p/miR-152-3p against ischemic injury by exacerbating inflammatory cascades and apoptosis regulatory mechanisms (Sun et al., 2023). Experimental data further demonstrate that XSB ameliorates BCAS-induced cognitive impairment via multimodal mechanisms including cerebral perfusion modulation, white matter lesion attenuation, glial cell activation suppression, and neuroinflammatory resolution. Gastrodin exerted neuroprotective effects in rats with neuroinflammation by impacting the TLR4-NF-κB-NLRP3 pathway. Therefore, gastrodin may be a potential therapeutic agent for neuroinflammation-induced cognitive dysfunction (Luo et al., 2024). By preventing brain endothelial injury, which preserves BBB integrity, reduces neuroinflammatory responses, and maintains the functional competence of the NVU, thrombin inhibition or targeting its downstream signaling effectors could offer a viable therapeutic approach for mitigating diabetes-associated dementia (Vittal Rao et al., 2021). Crucially, NF-κB signaling emerges as a central mediator in this pathological cascade (Xiao et al., 2024). The systemic inflammatory response exhibits dynamic characteristics, with cytokine profiles demonstrating temporal and severity-dependent variations in VaD. Age-related oxidative stress, persistent DNA damage accumulation, and compromised DNA repair mechanisms collectively jeopardize both neuronal genomic stability and the functional integrity of neurovascular components including endothelial cells, astrocytes, and pericytes (Li et al., 2019). Endothelial colony forming cells (ECFCs), a specialized subset of endothelial progenitors, play indispensable roles in vascular homeostasis, regenerative repair, and neovascularization through their clonal expansion potential. The aging process compromises ECFCs functionality via multifactorial pathways encompassing oxidative damage, sustained low-grade inflammation, and cellular senescence, ultimately manifesting as diminished vascular regenerative capacity and compromised neurovascular compliance. These progenitor cells directly modulate critical cerebrovascular functions such as NVC dynamics, BBB maintenance, and angiogenic processes. Progressive age-dependent depletion of ECFCs populations, coupled with functional deterioration, induces cerebral microvascular attrition and hemodynamic insufficiency, synergistically accelerating cognitive impairment progression (Negri et al., 2025).

**Figure 3:**
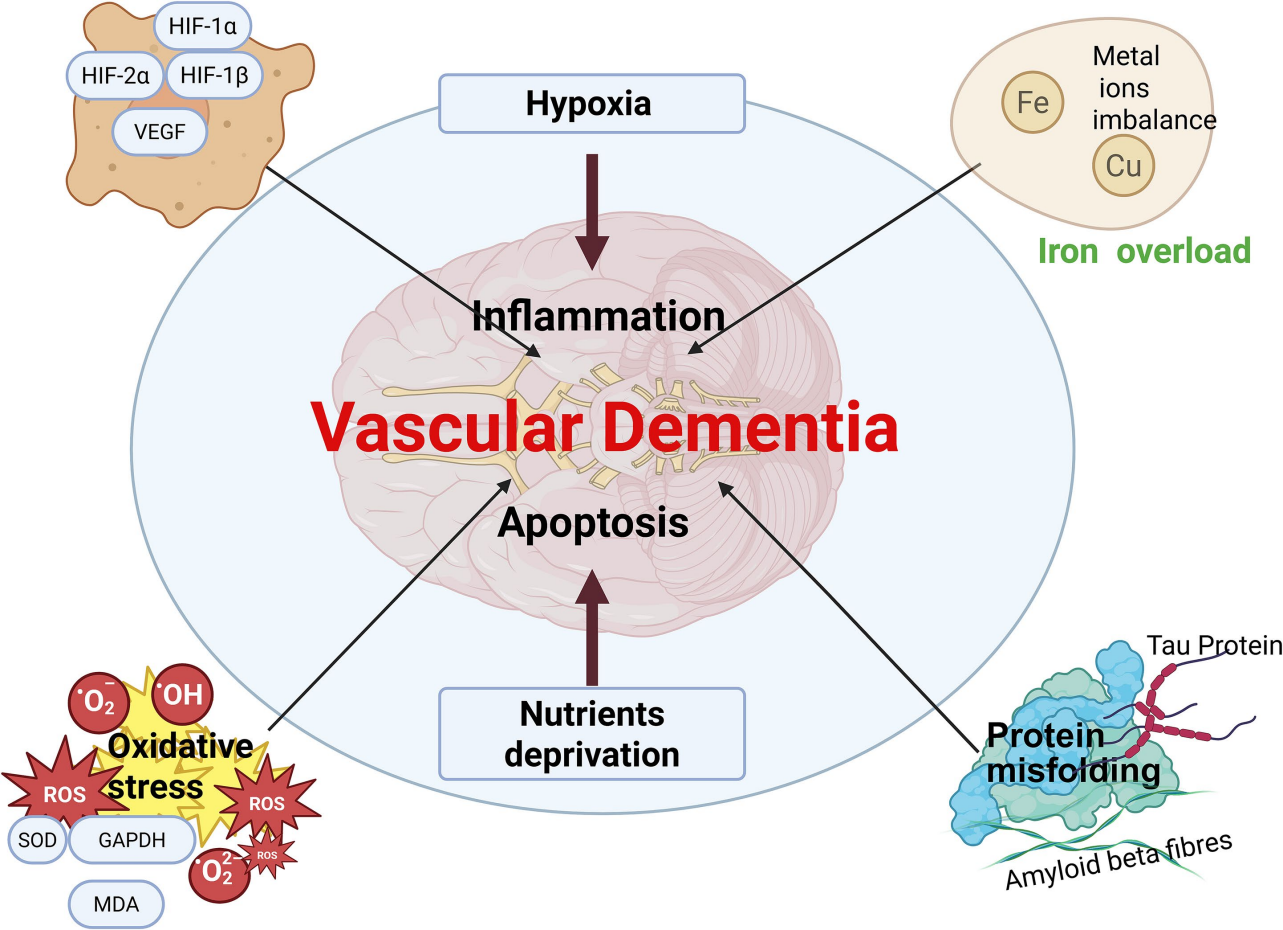
Schematic representation of hypoxia-inflammation-apoptosis in VaD. Hypoxia, inflammation, and apoptosis converge to induce neuronal loss, protein misfolding, ion imbalance, and the generation of reactive oxygen species, thereby exacerbating or contributing to the pathological damage observed in VaD. Created in https://BioRender.com.

The pathogenesis of VaD is highly complex, involving intricate interactions among various influencing factors. The “hypoxia-oxidative stress-inflammation” cascade forms a detrimental feedback loop, wherein the activation of one factor can trigger the others, ultimately resulting in progressive brain dysfunction during the course of vascular dementia. As depicted in Figure 3, hypoxia, oxidative stress, neuroinflammation, and apoptosis compromise the integrity of tight junctions, thereby inducing BBB disruption and subsequent brain injury.

### 3.4 Mitochondrial dysfunction leads to cerebrovascular endothelial cell injury

**Figure 4:**
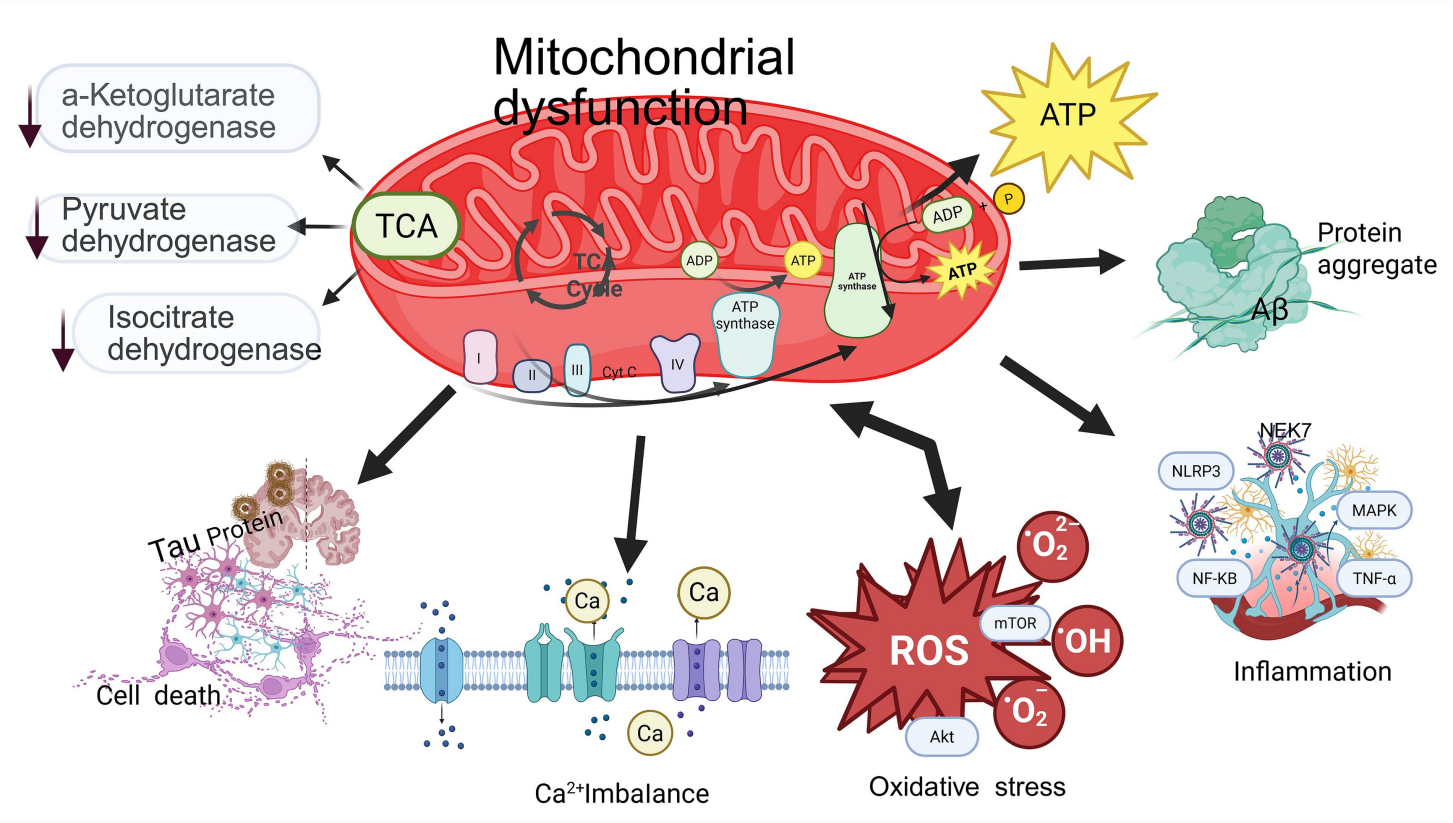
Schematic representation of the role of mitochondrial dysfunction in VaD. Mitochondrial dysfunction results in the dysregulation of the tricarboxylic acid (TCA) cycle, characterized by decreased activity of three key enzymes, excessive production of ROS, reduced ATP synthesis, and an imbalance in energy homeostasis. This mitochondrial impairment also induces oxidative stress, inflammatory responses, intracellular and extracellular Ca^2+^ imbalances, partial protein misfolding, and ultimately leads to apoptosis. Created in https://BioRender.com.

Substantial evidence has established the involvement of mitochondrial dysfunction in neurodegenerative disorders (Khacho et al., 2019; Wallace, 2005). Mitochondrial impairment constitutes a pivotal pathogenic mechanism underlying vascular dementia. Experimental findings indicate that gastrodin ameliorates CCH-induced mitochondrial dysfunction in VaD through modulation of the SIRT3/TFAM signaling axis, presenting a natural therapeutic target for dementia reversal (Chen et al., 2024). As demonstrated in Figure 4, integrated *in vivo* and *in vitro* investigations revealed that nicotinamide adenine dinucleotide (NAD^+^) administration attenuated CCH-induced cognitive impairment by suppressing ROS generation through SIRT1/PGC-1*α* pathway activation (Zhao et al., 2021). The AKT/mTOR signaling cascade, widely recognized as a canonical regulator of cellular homeostasis and bioenergetic metabolism, has emerged as a promising therapeutic focus for neurodegenerative disease intervention (Huang J. et al., 2022; Stiles, 2009). The AKT/mTOR signaling pathway has been demonstrated to play a pivotal regulatory role in mitochondrial homeostasis. Pharmacological modulation of S1PR2 substantially attenuates AKT/mTOR pathway activation, consequently suppressing A*β*_25-35_-induced autophagic flux in cellular models and ameliorating cognitive deficits (Fan et al., 2015). This neuroprotective mechanism may be mediated through the diminution of mitochondrial Ca^2+^ overload, which critically compromises mitochondrial bioenergetics (Zhou and Tan, 2020). Sirtuin 3 (SIRT3), a mitochondrial-localized NAD^+^-dependent deacetylase, serves as a master regulator of mitochondrial integrity through its governance of ATP biosynthesis, redox balance maintenance, and modulation of apoptotic or proliferative pathways. Emerging evidence highlights the pathogenic implications of SIRT3 dysregulation in neurodegenerative disorders. Therapeutic targeting of TDP43-driven Glycogen synthase kinase-3*β* (GSK3*β*) activation to restore ER-mitochondrial cross-talk represents a promising strategy with potential translational applications (Matsuyama et al., 2020). Free radicals originating from mitochondrial dysfunction contribute to homeostatic imbalance and lipid peroxidation. NOX4 exacerbates mitochondrial metabolic impairments and diminishes intracellular iron concentrations in patients via oxidative stress-mediated lipid peroxidation (Park et al., 2021). The augmentation of ROS generation through mitochondrial pathways, coupled with the activation of mitochondrial oxidative stress, suppression of ATP biosynthesis, and elevation of glycolytic activity, collectively inflict damage upon mitochondrial DNA. These mechanisms ultimately attenuate mitochondrial dysfunction and vascular inflammation within cerebral microvascular endothelial cells (Nyunt et al., 2019). In the context of traumatic brain injury, epileptic seizures and widespread cortical depolarization may potentiate secondary cerebral damage, precipitating mitochondrial dysfunction, compromised cortical perfusion, and neurobehavioral impairments, mitochondrial dysfunction underlies impaired NVC following traumatic brain injury (van Hameren et al., 2023).

### 3.5 The disruption of metal ion homeostasis contributes to cerebrovascular endothelial cell damage

Metal ion homeostasis plays a pivotal role in preserving normal physiological functions of organisms. Experimental evidence indicates that amorphous selenium substantially enhances learning and memory capacity in rodent models of VaD, promotes restoration of cerebral arterial hemodynamics, facilitates morphological recovery of hippocampal CA1 pyramidal neurons through dendritic remodeling, attenuates oxidative stress biomarkers, upregulates CaMKII protein expression, and effectively modulates intracellular calcium homeostasis (Zhu et al., 2023). In this investigation, we developed a novel metal-chelating compound that demonstrated potent inhibitory effects on Tau phosphorylation via iron ion sequestration both *in vitro* and *in vivo*, effectively alleviating pathological manifestations. These discoveries elucidate previously unrecognized mechanisms by which Tau protein aggregation mediates memory impairment (Zuo et al., 2024). Dietary exposure to elevated aluminum concentrations whether through food additives or contaminated water sources may exacerbate susceptibility to cognitive decline, with aluminum ions potentially functioning as synergistic participants in neurodegenerative cascades culminating in cerebral dementia (Solfrizzi et al., 2006).

Elevated intracellular iron ion concentrations induce substantial generation of ROS radicals, exacerbating oxidative stress and consequently precipitating cellular damage and apoptosis. Iron deposition in hippocampus in model groups which accompanied the decline of learning and memory function, iron deposition increased AMPK/autophagy pathway associated molecules in the hippocampus and promoted neuronal apoptosis, which might be a new pathogenesis in VaD (Huo et al., 2019). Copper ranks as the third most prevalent trace element in the cerebral environment, whereas zinc–an essential micronutrient released via synaptic vesicles during neuronal activation–serves a pivotal regulatory function in neural signal processing. Synaptic Cu^2+^ demonstrates synergistic coordination with Zn^2+^, potentiating neuronal degeneration and substantially contributing to the etiological mechanisms underlying VaD (Tanaka and Kawahara, 2017). While Mn^2+^ and Ni^2+^ exhibit modulatory effects on Zn^2+^-mediated neurotoxicity, their pathogenic influence remains notably diminished compared to Cu^2+^. Subthreshold Cu concentrations paradoxically amplify Zn-associated neurotoxic manifestations. Under pathological states including transient ischemic episodes, disrupted copper ion homeostasis culminates in aberrant Cu^2+^ accumulation, thereby facilitating excessive Zn^2+^ exocytosis into synaptic spaces and subsequent neuronal mortality (Kawahara et al., 2024).

## 4 Strategies and therapeutic approaches for the management of VaD

Given the accelerating trend of global aging and the rising prevalence of cerebrovascular pathologies, VaD has emerged as a critical public health challenge. Defined as a clinical syndrome manifesting cognitive impairment secondary to cerebrovascular disorders, encompassing ischemic stroke, hemorrhagic stroke, or chronic cerebral hypoperfusion, VaD demonstrates distinctive clinical characteristics and is widely recognized as “the sole modifiable form of dementia.” Early diagnosis significantly enhances the potential for mitigating dementia progression. The principal therapeutic objective involves secondary prevention of cerebrovascular events. Contemporary management strategies employ multidimensional interventions integrating preventive protocols with therapeutic modalities, addressing three key domains: vascular risk factor modification, pharmacological intervention, and non-pharmacological rehabilitation. Current evidence indicates that donepezil demonstrates limited efficacy (30–40% incidence rate), yielding marginal improvements in cognitive domains while exhibiting negligible therapeutic impact on functional capacities and global clinical outcomes. Adverse event profiles reveal notable incidence rates of nausea (20%), vomiting (15%), bradycardia (5%), and syncope (2%), with heightened susceptibility observed in geriatric populations. The disease’s clinical heterogeneity poses substantial therapeutic challenges, treatment alone approaches prove inadequate for addressing diverse pathophysiological subtypes and complicate treatment response predictability. Furthermore, non-pharmacological interventions face evidence-based limitations, including insufficient data from randomized controlled trials with extended follow-up periods, ambiguous mechanisms underlying traditional Chinese medicine applications, and inadequate standardization impeding clinical scalability.

Vascular dementia, classified as a potentially preventable and treatable form of dementia, stems from both the inherent complexity of the pathological process and the progressive evolution of underlying vascular pathologies including hypertension and diabetes mellitus. These mechanisms collectively precipitate recurrent cerebrovascular insults and exacerbate cognitive deterioration. The persistent cerebral hypoperfusion and metabolic derangements associated with VaD remain refractory to reversal through contemporary therapeutic modalities. Early clinical manifestations, typified by subclinical memory deficits and impaired executive functioning, frequently elude detection and are erroneously attributed to normative aging processes. Diagnostic recognition often occurs beyond the therapeutic window of maximal efficacy, compounded by the absence of validated sensitive biomarkers. Confronting these clinical challenges, the development of multi-target interventional paradigms and novel therapeutic frameworks is anticipated to constitute a predominant focus of translational research endeavors in this domain.

To investigate the potential therapeutic drugs and targets for the intervention and treatment of VaD from the perspective of microvascular endothelial cells. Table 1 summarizes target drugs and molecular compounds that enhance cognitive function by addressing vascular endothelial cell-related mechanisms through three key approaches: preserving BBB integrity, modulating NVC and CBF regulation, and utilizing targeted drug delivery systems. These strategies hold promises as novel preventive and therapeutic targets for VaD in the future.

**Table 1:** Target drugs that protect vascular endothelial cells to improve neurological function and cognitive impairment.

Categories	Drugs name	Mechanisms involved	Clinical research description	Whether to reverse vascular injury	References
	1. Polyphenols	It acts on brain endothelial cells to improve BBB integrity and reduce inflammation.	High intake of specific polyphenols, including flavonoids and phenolic acids, may help preserve verbal memory, a prominent area of vulnerability in pathological brain aging.	No, experimental basic research. It has potential application value.	Grabska-Kobyłecka et al. (2023)
Protect the integrity of the BBB	2. ASK1-K716R	It preserves BBB integrity by inhibiting ASK1/JNKs pathway in endothelial cells, consequently reducing the degradation of tight junction proteins.	No clinical data were available	No, the current research is experimental basic research. It has potential application value.	Meng et al. (2023)
	3. Serum exosomes	exosomes isolated from healthy serum provided neuroprotection against experimental stroke partially via inhibition of endothelial cell apoptosis and autophagy-mediated BBB breakdown.	ChiCTR1900021544	No, Intravenous serum-derived exosome treatment may, therefore, provide a novel clinical therapeutic strategy.	Huang L. Y. et al. (2022)
	4. Caveolin-1	They increased Cav-1 in the brain endothelium and increased transcellular BBB permeability to albumin, reduced paracellular BBB Claudin-5 tight junctions, and caused T-cell infiltration in the hippocampus.	No clinical data were available	No, the current research is experimental basic research. It has potential application value.	Trevino et al. (2024)
	5. Ubiquinol	plasma from Ub-supplemented patients improved the viability of endothelial cells.	ACTRN12619000436178	No, has experimental basic research.	García-Carpintero et al. (2021)
	6. Inhibition of piezo1	piezo1 might be involved in CCH-induced cognitive impairment and BBB damage.	No clinical data were available	Yes, has experimental basic research. CCH model was established by BCAS in rats and by (OGD/R) in bEnd.3 cells.	Xu et al. (2024)
	7. GSK3β Substrate-competitive Inhibitors	The GSK3β inhibitors TFGF-18 and ISO modulate the gut homeostasis and barrier function to inhibit neuroinflammation and attenuate cognitive impairment by regulating NF-κB, and Nrf2/HO-1 pathways.	No clinical data were available	No. Glycogen synthase kinase 3 (GSK3β) is a potential therapeutic target.	Zhang et al. (2024)
NVC Adjustment of CBF	1. Nicotinamide mononucleotide	NMN supplementation rescued the NVC response by increasing endothelial NO-mediated vasodilation, which was associated with significant improvements in spatial working memory and gait coordination, expanding the CBF response.	ClinicalTrials.gov NCT04823260 Clinical Trial Registry-India, CTRI/2021/03/032421	No, the cerebro microvascular protective effects of NMN highlight the preventive and therapeutic potential of NAD intermediates as effective interventions in patients at risk for VCI.	Tarantini et al. (2019)
	2.mitochondrial-targeted antioxidant peptide SS-31	Treatment with SS-31 significantly improved NVC responses by increasing NO-mediated Cerebro microvascular dilation, which was associated with significantly improved spatial working memory, motor skill learning, and gait coordination.	No clinical data were available	No, we propose that mitochondria-targeted antioxidants may be considered for pharmacological microvascular protection for the prevention/treatment of age-related VCI.	Tarantini et al. (2018)
	3. MCC950	NLRP3 inhibition with MCC950 will improve NVU remodeling and cognitive outcome following stroke via vagotropic uncoupling between endothelial cells and hippocampal neurons.	No clinical data were available	No, MCC950 directly interacts with the Walker B motif within the NLRP3 NACHT domain, thereby blocking ATP hydrolysis and inhibiting NLRP3.	Ward et al. (2019)
	4. PI3K block	PI3K, an enzyme that diminishes the bioavailability of PIP^+^_2_ by converting it to phosphatidylinositol (3, 4, 5)-trisphosphate (PIP3), restored Kir2.1 channel activity, capillary-to-arteriole dilation, and functional hyperemia.	No clinical data were available	No, PI3K inhibition is a viable therapeutic strategy for treating defective NVC and cognitive impairment associated with cSVD.	Thakore et al. (2023)
	5. Resveratrol	It rescues cortical NVC responses to increased neuronal activity in aged mice, likely by restoring Cerebro microvascular endothelial function via downregulation of NADPH oxidase-derived ROS production. Beneficial Cerebro microvascular effects of resveratrol may contribute to its protective effects on cognitive function in aging.	ClinicalTrials.gov IDNCT01794351	No, the current research is experimental basic research. It has potential application value.	Toth et al. (2014)
	6. Taxol	In PTX treated mice, both treatments effectively eliminated senescent endothelial cells, rescued endothelium-mediated NVC responses and BBB integrity, increased capillarization and improved cognitive performance.	ClinicalTrials.gov IDNCT02753036	No, senolytic treatments can be a promising strategy for preventing chemotherapy-induced cognitive impairment.	Ahire et al. (2023)
	7. ABT263/Navitoclax	The neurovascular protective effects of ABT263/Navitoclax treatment highlight the preventive and therapeutic potential of senolytic treatments effective interventions in patients at risk for vascular cognitive impairment VCI.	No clinical data were available	No, the neurovascular protective effects of ABT263/Navitoclax treatment highlight the preventive and therapeutic potential of senolytic treatments (as monotherapy or as part of combination treatment regimens) as effective interventions in patients at risk for VCI.	Tarantini et al. (2021a)
Targeted drug delivery systems	1.containing fusogenic liposome (FL-RSV)-based molecular delivery system	FL-RSV rescues endothelial function and NVC responses in aged mice.	No clinical data were available	No, resveratrol containing fusogenic liposomes could also be used for combined delivery of various anti-gerontic factors, targeting key pathways involved in microvascular aging and neurovascular dysfunction for the prevention/treatment of age-related Cerebro microvascular pathologies and development of VCI in aging.	Wiedenhoeft et al. (2019)
	2. Nano-delivery systems for encapsulation of dietary polyphenols	It provide an overview of the primary polyphenols used for ND and brain tumor prevention and treatment by focusing on recent findings, the principal factors.	No clinical data were available	No, It has potential application value.	Squillaro et al. (2018)
	3. Angiopep-2 liposomes (Ang-Sal/Ica-Lip)	It could reverse neuronal and synaptic damage, inhibit neuroinflammation and oxidative stress and improve learning and cognitive function.	No clinical data were available	No, it may be a promising therapeutic strategy for mitigating related symptoms.	Zhang et al. (2023)
	4. Ang2-ICA/TSIIA liposomes	It could improve AD-like pathological features in APP/PS1 mice, including inhibiting neuroinflammation and oxidative stress, reducing apoptosis, protecting neurons, and improving cognitive function.	No clinical data were available	No, Ang2-ICA/TSIIA liposomes are considered a potentially effective therapeutic strategy for AD. It has potential application value.	Wang et al. (2022)
	5. ApoE (ApoE-Res/Sal-Lips)	The ApoE-Res/Sal-Lips were found to alleviate AD pathological symptoms, reduce learning and memory impairments, and improve brain function.	No clinical data were available	No, has experimental basic research. ApoE-Res/Sal-Lips provide a new method for the treatment of AD.	Chen et al. (2023)

With the in-depth investigation into the pathological mechanisms of VaD and advancements in emerging technologies, research is being conducted to develop new drugs targeting these mechanisms. These efforts aim to enhance vascular stability, promote angiogenesis, stimulate neural regeneration, and improve synaptic plasticity. Dual protection of both the vascular and neural systems is achieved through genetic screening, precision medicine-based diagnosis, and personalized adaptive cognitive training utilizing digital therapy and telemedicine. Additionally, deep brain stimulation of specific nuclei such as the anterior thalamic nucleus, and techniques to strengthen neural network connections. The treatment paradigm for VaD is transitioning from “single symptomatic treatment” to “multi-target intervention” and from “disease treatment” to “early prevention and repair.” Through clinical trials focusing on VaD-related strategies and methods involving brain microvascular endothelial cells, early intervention approaches are being refined, and comprehensive interventions addressing vascular risk factors alongside cognitive training are being implemented to delay the progression of dementia. It is anticipated that these efforts will significantly enhance the prognosis and quality of life for VaD patients in the future. Table 2 Data from https://clinicaltrials.gov/ (June 13, 2025) database. Cognitive behavioral training was implemented through structured exercise regimens, followed by neuroradiological imaging for preliminary lesion diagnosis. To enhance prognosis, Equol and DL-NBP supplementation were administered to mitigate dementia symptoms. It should be noted that these methodologies currently remain experimental; however, they demonstrate potential clinical utility pending further validation.

**Table 2:** Clinical research strategies to delay the development of vascular dementia.

Drug name/Strategy approach	Mechanism of action	Clinical trials research progress	NCT number	Study type	Study sites	References
DL-NBP	Effects on cognitive function and vascular regulation in patients with mild VaD caused by subcortical ischemic vascular disease.	ClinicalTrials.gov ID Not Applicable	NCT03906123	Interventional	Tianjin, China MUGH001	Feng et al. (2023) and Feng et al. (2021)
Multicomponent training (MT)	A combination of aerobics, strength, balance, and posture exercises may be effective training to improve cognitive function in dementia.	ClinicalTrials.gov	NCT04095962	Interventional	Portugal BB01	
CADASIL (Diagnosis)	Laboratory methods designed to distinguish progenitors of specific cell populations (endothelial and vascular smooth muscle cells) from peripheral-blood mononuclear cells (PBMC) or skin fibroblasts. Plasma components were isolated from peripheral blood for study and characterization of potential circulating biomarkers.	ClinicalTrials.gov ID Observational studies are ongoing	NCT06938100	Observational	Italy, Spain GENICa	Markus (2023) and Yuan et al. (2024)
Equol	supplement Equol can slow the progression of arteriosclerosis, cerebral small vessel disease, and memory decline. Plasma biomarkers of inflammation and endothelial function, such as reactive proteins, and vascular cell adhesion molecules, were measured.	ClinicalTrials.gov ID Phase2	NCT05741060	Interventional	America STUDY22010165	Sekikawa et al. (2022)

## 5 Perspective outlook

Vascular dementia, currently regarded as a contentious entity within dementia classification systems, poses significant therapeutic challenges. From my point of view, early pharmacological intervention at the incipient phase of cognitive impairment may arrest disease progression and potentially reverse pathophysiological alterations. A critical barrier in therapeutic development lies in the paucity of validated molecular targets for VaD, though emerging methodologies in systems biology and multimodal data integration are progressively addressing this knowledge gap. To establish precision medicine paradigms, comprehensive meta-analyses of longitudinal clinical cohorts coupled with histopathological validation through stereotactic brain tissue sampling are imperative for target identification. Central to VaD pathogenesis is cerebrovascular endothelial dysregulation, which orchestrates neuronal degeneration and cognitive deterioration via interdependent mechanisms: BBB compromise, NVU, redox imbalance, and glial-mediated neuroinflammation.

Vascular injury is a key cause of neuronal loss and synaptic disintegration. The innate immune cells, pattern recognition receptors, related inflammatory mechanisms disrupt cerebrovascular integrity via glial activation and increased pro-inflammatory interleukins and TNF-*α*. Inflammasome polymorphisms and multi-faceted neuro-immune interactions further integrate systemic and central inflammatory pathways, which induce vascular tissue injury and neurodegeneration. Given the multifactorial pathogenesis of vascular dementia and extensive research findings, monotherapeutic approaches are unlikely to address all disease stages effectively. Current evidence suggests that optimal therapeutic strategies should adopt multi-target combination therapies targeting key molecular pathways. This may involve synergistic integration of antioxidant agents, anti-inflammatory compounds, and cerebral perfusion-enhancing interventions to comprehensively modulate the complex pathophysiological cascade. To promote the restoration of homeostatic balance in cerebrovascular endothelial cells and ameliorate neurological deficits through targeted interventions addressing distinct pathophysiological mechanisms. Future investigations delineating the pathophysiological mechanisms underlying cerebral microvascular endothelial dysfunction in VaD will advance our understanding of disease pathogenesis and potentially inform the development of novel therapeutic strategies for this debilitating condition.
